# Intelligence

**Published:** 1827-02

**Authors:** 


### INTELLIGENCE.

### MONTHLY REPORT OF PREVALENT DISEASES.

The only object we have in view in these Reports is to give an idea of those diseases which are most prevalent. During the past month, however, nothing has occurred which appears worthy of being recorded, except that the fever mentioned on former occasions seems decidedly to have decreased. We are not aware that there exists at present any disease which is especially entitled to be called prevalent, beyond the catarrhal affections and rheumatism which always abound at this season.

*January* 25*th*.

### BILLS OF MORTALITY FOR 1826.

### DISEASES.

| | | | | | |
|---|---|---|---|---|---|
| Abscess | 76 | Fever | 926 | Palsy | 22 |
| Age, and Debility | 1575 | Fever, Typhus | 97 | Paralytic | 125 |
| Apoplexy | 363 | Fever, Intermittent, or Ague | 2 | Pleurisy | 13 |
| Asthma | 922 | Flux | 8 | Rheumatism | 12 |
| Bedridden | 1 | Gout | 38 | Scrofula | 10 |
| Bile | 14 | Hemorrhage | 34 | Small-Pox | 503 |
| Cancer | 100 | Hernia | 28 | Sore Throat, or Quinsey | 13 |
| Childbirth | 209 | Hooping Cough | 674 | Spasm | 44 |
| Consumption | 5290 | Hydrophobia | 4 | Stillborn | 733 |
| Contraction of the Heart | 2 | Inflammation | 2295 | Stone | 21 |
| Convulsions | 2588 | Inflammation of the Liver | 119 | Stoppage in the Stomach | 20 |
| Croup | 90 | Insanity | 170 | Suddenly | 110 |
| Diarrhœa | 12 | Jaundice | 33 | Teething | 309 |
| Dropsy | 820 | Jaw-locked | 1 | Thrush | 65 |
| Dropsy on the Brain | 676 | Measles | 774 | Tumor | 8 |
| Dropsy on the Chest | 65 | Miscarriage | 3 | Venereal | 8 |
| Dysentery | 7 | Mortification | 244 | Worms | 1 |
| Enlargement of the Heart | 16 | Ossification of the Heart | 6 | Total of Diseases | 20374 |
| Epilepsy | 40 | Palpitation of the Heart | 6 | | |
| Eruptive Diseases | 12 | | | | |
| Erysipelas | 17 | | | | |

### CASUALTIES.

| | | | | | |
|---|---|---|---|---|---|
| Burnt | 28 | Frighted | 1 | Smothered | 1 |
| Choked | 1 | Killed by Falls and several other accidents | 112 | Starved | 2 |
| Drowned | 139 | Murdered | 4 | Suffocated | 8 |
| Excessive Drinking | 7 | Poisoned | 8 | Suicides | 57 |
| Executed | 2 | Scalded | 2 | Total of Casualties | 384 |
| Found dead | 9 | Shot | 1 | | |
| Fractured | 2 | | | | |

Christened....Males, 11,178—Females, 11,066—In all, 22,244.
Buried ......Males, 10,454—Females, 10,304—In all, 20,758:

Whereof have died—

| | | | |
|---|---|---|---|
| Under Two Years of Age | 5952 | Sixty and Seventy | 1832 |
| Between Two and Five | 1982 | Seventy and Eighty | 1569 |
| Five and Ten | 768 | Eighty and Ninety | 634 |
| Ten and Twenty | 808 | Ninety and a Hundred | 90 |
| Twenty and Thirty | 1472 | A Hundred | 1 |
| Thirty and Forty | 1724 | A Hundred and Three | 3 |
| Forty and Fifty | 1994 | A Hundred and Five | 3 |
| Fifty and Sixty | 1926 | | |

Decreased in the Burials this year, 268.

☞ There have been executed within the Bills of Mortality, 19; of which number only 2 have been reported as such.

---

*Case of Uterine Hemorrhage, successfully treated by the Operation of Transfusion.*
By Burton Brown, Esq.

Mrs. ——, ætatis thirty, was delivered of her tenth child, on the 31st December, at a quarter before one P.M. This lady had, in all her preceding labours, suffered much from uterine hemorrhage; she was of a lax and delicate habit, and hysterical in the highest degree. Soon after the birth of the child, an alarming flooding occurred: the hand was immediately introduced into the uterus, for the purpose of exciting contraction; which effect was almost instantly produced, the womb expelling both the hand and placenta into the vagina, and from this period the hemorrhage ceased. Although I have seen much of flooding, yet the symptoms of collapse were such in the present instance as to produce in my mind a considerable degree of anxiety and alarm. I administered from time to time small portions of pure brandy, amounting in the whole to eight or ten ounces; in addition to which she took a draught composed of Ether and Camphor Mixture. It should perhaps be stated, that, in addition to the introduction of the hand within the uterine cavity, cold was extensively applied to the abdomen, and the head was lowered. The uterus continued very firmly contracted, except at intervals, when there was a slight relaxation for a few seconds,—not, however, sufficient to allow of the escape of any blood from the uterine vessels. The patient had three smart convulsive fits, each followed by a very alarming collapse. As she appeared to have slightly rallied in consequence of the brandy and ether, I contented myself for a short time with watching her very closely.

She now vomited, and, as frequently happens in these cases, appeared to be a little roused by it; but her pulse soon flagged again, the breathing became irregular and weaker, and from this time the powers of deglutition were lost. She became now perfectly insensible; the head was thrown back, the eyelids closed, and the pupils fully dilated. The pulsation of the carotid artery was just perceptible, and the radial artery imparted to the finger only a diffused tremor.

I at this period requested the assistance of Dr. Blundell, who was from home, as was also Mr. Doubleday.* Mr. Waller, of Aldersgate-street, was now sent for, and arrived about half-past two, (an hour and three-quarters after the birth of the child,) when the patient was in the following state:—She was lying on her back, with a most death-like expression of countenance; her extremities were of a marble coldness, and there was but little warmth on her chest or abdomen. She had, in a most remarkable degree, the high respiration, which was accompanied with a good deal of stertor. The eyelids were closed, and the eyes perfectly insensible to light; pupils fully dilated. The jaw was dropped. The pulse at the wrist was not to be felt; nor could Mr. W. distinguish the beat of the carotid. It was my firm conviction at this period that she would not survive long enough to have the operation of transfusion performed, though, after a deliberate consultation upon the case, we determined to make the attempt, not considering ourselves justified in

* This gentlemen arrived, however, at the termination of the second injection.

omitting it, even under the present discouraging circumstances. The operation was proceeded in after the usual manner.

Twenty-five minutes before three o'clock, (two hours and ten minutes after the birth of the child,) thirteen drachms of blood were injected through the median vein, in a direction towards the heart, by means of a syringe; the blood remaining out of the living body, on the whole, about a minute and a half. No particular change seemed to be produced by this first supply of blood, which was injected with the greatest ease, and no unpleasant symptoms followed it. Care was of course taken to prevent the admission of air, and the blood was thrown in very slowly.

Five minutes having elapsed, the injection of thirteen drachms was repeated, in the same slow and cautious manner; and now the action of the radial artery became decidedly improved: it was no longer a mere tremulous flutter, but a distinct pulsation. The respiration soon became more free. The pupil was still exceedingly dilated, and no signs of sensibility were manifest; the palpebræ were of a mottled hue, and the countenance extremely pallid.

Ten minutes having elapsed from the second injection, the operation was repeated, and the improvement was now more evident, (the aggregate quantity injected amounting to about five ounces.) The pulse was 120, and regular. Mr. Doubleday informed me that, at the time of the third injection, he separated the palpebræ, and passed the point of his finger several times before the cornea, but no sensibility was manifested, either by motion of the globe or contraction of the iris. She, however, moved her extremities, and exclaimed "Oh!" The countenance was less pale; she sighed deeply, and breathed with a more expanded thorax. About three tea-spoonfuls of brandy were now given her, mixed with a little water, which she swallowed with tolerable ease, being put into her mouth by a spoon.

It was not, however, till after the fourth injection that the amelioration was most conspicuous. Now the pupil contracted readily upon the admission of light; she moved her extremities frequently, and with considerable power; the chest was fully expanded at each inspiration; pulse 120, equable, and moderately forcible. She appeared to have much pain or uneasiness in her left side, evinced by some restlessness, by putting her hand to her side, and inclining the trunk laterally, as though she would shrink from herself; the corrugatores supercilii forcibly contracted. These indications of the existence of pain were particularly observable when pressure was made on the uterine region. The cheeks became reddened during the contraction of the facial muscles while in pain, and the lips, from the powerful action of the orbicularis cris, were quite florid.—Were these the effect of after-pains? I think she recognised her husband when he spoke to her, and she grasped my hand as though she knew me.

It was now evident that the threatening symptoms were subdued, but it became a question whether another injection would not tend to alleviate the unpleasant symptoms which so often follow uterine hemorrhage, even when patients eventually do well; and consequently a fifth was attempted, but, owing to the patient's getting a little restless, and some other cross incidents, it was abandoned. When the pressure was removed from the lower part of the vein which had been opened, the blood issued in a small jet; thus proving, beyond controversy, the complete restoration of the circulation.

She now directed her eyes towards surrounding objects, and attempted to adjust her dress. An hour and ten minutes after the first injection, she noticed all around her; she appeared soothed by the attention of her nurse, and spoke to me with a firm, distinct voice, desiring that I would not leave her. She appeared to have much nervous irritation, or hysterical mobility; to alleviate which, fifty drops of Tinct. Opii were given. At a quarter to four, she turned upon her side: this was three hours and a half after the birth of the child.

Half-past six.—Has had no sleep. Great uneasiness and heat of the head. She had taken toast and water, and diluted milk. An evaporating lotion was directed to be frequently applied to the head, and a draught composed of Liq. Am. Acet. and Mist. Camph., with twenty minims of Tr. Hyoscyam. to be taken every six hours.

January 1st, ten P.M.—Had taken light nourishment, such as egg beat up with milk. Half an ounce of Castor-oil was given early in the morning, which produced a copious evacuation. In the evening, she complained of intense pain in the head. Pulse equable, and moderately firm; much heat in the head and temples; skin and extremities ordinarily warm. Diet during the day has been chiefly milk, and the yolk of one egg. Respiration free and easy; sensibility perfect; in short, the headache was the only bad symptom.

2d, morning.—The Castor-oil was repeated, which again produced a plentiful evacuation. The pulse 100, strong and bounding; the surface of the body hot, but the heat and pain in the head somewhat diminished. Some interrupted sleep was obtained during the night. The mind remains collected and tranquil.

Afternoon.—Complains of acute pain in the forehead, principally over the left eye, extending down the side of the face, with occasional confusion. In the evening, the pain was less, and its occurrence not so frequent; temperature of the head lessened, and also that of the skin; pulse 100, equable and compressible.

3d, morning.—Passed a restless night; sleep disturbed; considerable pain in the head, with increased temperature; was constantly corrugating the skin of the forehead and eye-brows; had resisted the application of the lotion during the night. Complains of pain in the bowels, for which warm flannels were applied with benefit; and had passed three small, lax motions. Tongue cleaner, moist, and rather pale-looking; pulse 100, and strong.—To have six leeches applied to the temples.

Eight P.M.—Leeches bled well. Pain at the head less; heat reduced; tongue free from the creamy crust with which it was covered last evening, and this morning moist and looks pale; temperature of the skin natural; pulse 100, soft and compressible. The arm was dressed the first time; looked well; not any tenderness, or indication of inflammation along the course of the vein.—To take thirty drops of laudanum in one of the draughts at bed-time.

4th, ten A.M.—Was quieted by the opiate, though the sleep was disturbed. Pain in the head less severe, and not so frequent in its attacks. Her hair has been removed, from which she has found benefit. Lotion pleasant to the feel. Pulse 106, and soft; skin generally temperate; lochial discharge in sufficient quantity. Not any indication of lacteal secretion. To take ℥ ss. of Ol. Ricini, not having had stools since yesterday morning. Tongue coated with a brownish crust. Has taken a pint of milk in the twenty-four hours, besides the yolk of one egg, in addition to other diluents.

Nine P.M.—Had four stools. Tongue as morning; skin temperate; complains of cold generally; recurrence of pain in the head not so frequent.—To take the opiate draught at bed-time.

5th, ten A.M.—Had a restless night from hysterical affection; violent fits of crying; did not sleep till six in the morning. Pain in the head less; tongue coated as before; pulse 106, and soft; moist skin, not much above natural temperature.—Ordered the draughts to be continued, with the addition of twenty minims of Spirit. Æther. to each.

Evening.—Has complained of cold, and also pain in the right hip, extending down the outer side of the thigh; is very hysterical. Lochial discharge of good quality, and rather copious. Pulse eighty; tongue the same; skin temperate.—To take the opiate at night, and a Rhubarb draught in the morning.

6th, A.M.—Passed a restless night, apparently more from hysterical affection than any other cause. Other symptoms the same as last evening.—The Rhubarb draught ordered to be repeated, the first not having answered.

Evening.—Pulse seventy; tongue looking moist, and nearly free from crust; skin temperature; complained of pain extending from the spine of the ileum down the inner part of the right thigh, in the course of the crural nerve. Had passed two plentiful motions. Had used but little of the evaporating lotion, not requiring it.—To continue the draughts.

7th, A.M.—Had rested tolerably well, without the opiate draught, till three o'clock. Passed suddenly a large quantity of fresh-coloured lochial discharge, with some coagula, which alarmed her: she fainted, and, upon recovering, felt numbness in the hands. I was sent for at six o'clock, and found her looking pale and very restless, with considerable agitation; pulse 120, and small; tongue perfectly clean. Her sensorium was disturbed, and she had refused the application of the lotion to her head, which had been ordered to be applied tepid, since she had complained of the cold. Had taken plentifully of beef-tea. She complained of being much troubled with wind, and was constantly eructating. These symptoms I considered as obviously hysterical.—She was ordered to take thirty drops of laudanum immediately.

7th, eight P.M.—Has passed copiously fetid lochial discharge, with some coagula. Tongue clean and moist; skin cool; pulse eighty and small.—Ordered to take the opiate at bed-time.

From this time there were no particular unpleasant symptoms, except those of an hysterical nature, to which she was constitutionally subject.

*Stamford-street; January,* 1827.

---

*Vaccination.*—The Editor has received the following letter from Dr. GREGORY:

Sir,—Your last Number contains some critical observations, by Mr. NORTH, on the mode of vaccinating which I recommended in a former Number of your Journal. Were I to allow these remarks to pass unnoticed, I might, perhaps, by some of your readers, be considered as tacitly assenting to the correctness of his criticisms. As this is by no means the case, I trust you will favour me by the insertion of a few observations in reply.

Mr. North's principal objections apply, first, to my statement that the success of the operation is not influenced by the quantity of blood that flows from the incisions; secondly, to my recommendation of a very sharp lancet; thirdly, to my *dogma,* that the vaccine lymph is fully elaborated or developed by the fourth or fifth day, and is even then more intense than on the eighth day; fourthly, to my practice of making six or eight punctures. On each of these heads I have a few remarks to offer.

1. The question whether bleeding from the wounds be or be not indifferent to the success of the operation, may easily be decided by a few comparative trials; but Mr. North has *assumed* as an axiom, that by such bleeding the lymph is either washed away altogether or over diluted. I have yet to learn how these positions are *proved.* The fact that a pretty free flow of blood from the wounds does not always (or necessarily) prevent a successful result, is undeniable. I have witnessed it many times. If it should be ascertained that, *cæteris paribus,* the greater the bleeding the less the chance of success, still Mr. North's theory is doubtful; for hitherto it has not been decided how long the virus must remain in contact with the wounded surface to produce its effect,—whether seconds, minutes, or hours.

2. Mr. North states that "the very sharp lancets which I recommend are objectionable," and adds, "I will not say that Dr. Gregory is singular in this opinion, but I *know* that most practitioners prefer a lancet with a roundish and rather *blunt* point." Now, Mr. North has either had experience in the use of very sharp lancets, or he has not. If he has not, how is he enabled to speak so confidently of their injurious tendency; and if he has, what becomes of his inuendo, that this practice is peculiar to myself?

3. My "dogma," that "vaccine lymph is in a state of great perfection and high intensity when first formed," is not (according to Mr. North,) "in accordance with the perfect development and formation of other morbid poisons." I should be much gratified by learning what are the morbid poisons here specially referred to. I have always been led to believe that the matter of chancre, gonorrhœa, psora, and ophthalmia, is capable of propagating each respective disease from the very moment of its formation. If this opinion is erroneous, I should wish to learn how long after the first appearance of gonorrhœal running a patient may connect himself with women, without endangering their safety?

4. Mr. North objects to making six or eight punctures, having sometimes (though such cases, he allows, are *by no means common,*) seen "severe and unmanageable inflammation, and great general disturbance," arise from such a cause; whereas "troublesome symptoms rarely, *if ever,* occur from the insertion of two or three punctures." In reply to this I beg to observe, that local and constitutional disturbance accompanying vaccination appear to me to depend altogether upon the *habit* of the child; and if its system be heated, or predisposed to inflammation, such effects will follow, whether one, two, six, or ten punctures are made. The fault lies, not in the *number* of punctures, but in the period chosen for the performance of the operation. The same child, two months before, or two months after, might have had double the number of insertions made, without any unpleasant consequences, either local or general. Besides, if, with Mr. North, we look to the "development of other morbid poisons," we shall have still further reason to question the correctness of this criticism. In small-pox the practitioner would find but very little disturbance, either local or general, though the papulæ scattered over the body, or even collected on the face, were twice or even three times eight in number.

I have only further to add, that the mode of vaccination which I recommend is the result of considerable experience at the Small-Pox Hospital, where, during the year 1825, 4003, and in the year 1826, 3006 persons were vaccinated, making a total of 7019 persons who have been under my observation during the short period of two years.

I am, Sir, your very obedient humble servant,

*Jan.* 6, 1827. GEORGE GREGORY.

---

*Letter on the Subject of Vaccination from Mr. Greenhow.*

Sir,—Vaccination is a subject of so much essential importance to the safety and well-being of society, that every thing relating to its ultimate efficiency, or to the immediate success of the operation itself, cannot but possess considerable interest; and, certainly, if one mode of introducing the vaccine virus into the constitution is in reality more certain than another, it is important that this should be ascertained with precision, and well understood. It is in consequence of this consideration that I am induced to offer a few remarks on your correspondent's (Mr. North's) reply to Dr. Gregory's observations on the best mode of vaccinating.

Mr. North in the first place disagrees entirely with Dr. Gregory respecting the use of a sharp lancet in performing this simple, but important operation, and concludes that he does so in common with most practitioners. If, like myself, other practitioners have hitherto preferred a lancet "with a roundish and rather blunt point" from theoretical considerations, like those pointed out by Mr. North, without having tried the alternative of using a sharp one for the purpose, it becomes the more necessary that the question should be satisfactorily decided, and that, if in error, "most practitioners" should be set right on this essentially practical point. Since reading Dr. Gregory's paper in your Journal for November, I have myself tried a sharp lancet in vaccinating, and have certainly found its use more pleasant at the time, producing less irritation in the child, and more certain in its effect as regards the production of the disease. Its advantage over a blunt lancet seems to consist in its penetrating with sufficient ease to prevent the lymph from being rubbed off in passing through the cuticle, and entirely left on the surface, a cause of failure in operating with a blunt lancet which I am inclined to believe is by no means unfrequent. With regard to the quantity of blood drawn during the operation, I agree with Mr. North, and for the reasons he adduces, in thinking that its success is more certain when little, than when much, blood flows from the incisions. Whether using a sharp or a blunt lancet, I have ever avoided the discharge of much blood; and, if carefully used, this may be as certainly done with the former as with the latter instrument.

With respect to the number of incisions, there are many reasons which render it desirable that there should be several of them. 1. To increase the chances of success in communicating the disease. 2. To avoid the hazard of its progress being interfered with by the accidental injury of the pustule. 3. To obtain a supply of virus for future vaccination. And 4. To insure a characteristic scar being left on the arm sufficiently conspicuous and well defined to remove all future doubt of the individual having passed through the disease in a satisfactory manner. It seems to me a matter of indifference in what order the punctures may be arranged, provided they are made so distant from each other, as to avoid the danger of the pustules coalescing. I have been in the habit of making incisions in each arm, generally three in number, arranged longitudinally, thus ⋮; but, since reading Dr. Gregory's paper, I have tried the arrangement he recommends, and have found it answer well, in so far as it has left a most distinct indication of a regular and satisfactory disease. There is this advantage in introducing the lymph into both arms, that by watching the progress and decline of the disease we ascertain that it is the same in each, and have thus the best proof of the constitution being properly under its influence.

Provided the punctures are made at sufficient distances to prevent the coalition of the pustules, I should not fear too great a degree of inflammation from any number the operator may choose to insert. The only instances of inordinate inflammation from vaccination that have come under my notice have been produced by the confluence of several into one large and irregular pustule ; and in these cases I conceive the specific nature of the disease to have been destroyed. The inflammation spread extensively over the arm and breast, a sloughing abscess was the consequence, the life of the child was endangered, but not lost, and, although an unusually large and irregular scar remained, I must consider its protecting influence as at least extremely doubtful. It is from the hazard of the specific inflammation of cow-pox passing into that of a phlegmonous or erysipelatous nature, when several scratches or punctures are made in immediate contact, that this mode of operating, as employed by many practitioners, is to be objected to. But, in whatever way the vaccine lymph may be introduced into the human constitution, I am satisfied of the importance of repeatedly inspecting the progress of the disease throughout its course, until the separation of the crusts. It is in this way alone that an adequate knowledge can be obtained of its perfection, and of the degree of reliance to be placed upon it. Hence arises (as I have elsewhere* pointed out) the propriety of restricting the performance of vaccination to the members of the profession, and I am sure there cannot now be any need for the poor to apply elsewhere. There are medical gentlemen in every village who will not refuse to vaccinate gratuitously, if necessary, the children of their indigent neighbours.

I am, Sir, your very obedient servant,

*Newcastle-upon-Tyne ;* T. M. GREENHOW.
*Jan. 15th,* 1827.

---

### *Edinburgh Journal of Medical Science.*

" Mark now, how a plain tale shall put you down."—*Shakspeare.*

*New Species of " Amende Honorable."*—In the Number for October, 1826, of a new periodical, called the " Edinburgh Journal of Medical Science," were contained two papers, and two engravings, taken from the Medical and Physical Journal without acknowledgment. Of this plagiarism we complained in our Number for November; a few days after the publication of which, we received a note from the editors of the Journal in question, containing an apology for the " offence," and we found that it had been sent in consequence of the proprietors of this Journal having threatened them with an action at law in the event of the same conduct being repeated. The letter is as follows :—

" The Editor of the Edinburgh Journal of Medical Science has observed, with much regret, the offence, unintentionally given, by the omission of the name of the London Medical and Physical Journal to the articles of Messrs. Bell and Shaw. A second examination will shew the proprietors of the latter that the papers are curtailed and reduced, for a place in the ' Medical Intelligence' of the former publication; and that the transference of Mr. Shaw's case from the small type, in which references are scarcely ever made, was a mere matter of typographical arrangement, the control over which was for the time delegated to the printer's foreman. That no hostility was intended, may be seen by the allusion made to the Medical and Physical Journal in page 409 of the same Number ; and, it may be, that a wish to do no injustice to Mr. Shaw's instructive case was the reason for preferring a bad copy of his engraving to the many original drawings previously in the artist's hands. In fine, all the injustice done shall be repaid, at the price marked, in next Number ; and the Edinburgh Journal of Medical Science will take the threat of the Lord Chancellor's kind interference into the bargain."

" 62, *S. Bridge-street, Oct.* 29, 1826."

We were aware that this statement was not absolutely veracious, but still it appeared to be the apology of a man who was anxious to *back out* of the

* See my " Estimate of the True Value of Vaccination," &c. published about two years ago.

dilemma. In our Number for December, therefore, being the next after the above note was received, we inserted the following:—

"We have received a letter from the Editor of the Edinburgh Journal of Medical Science, and are perfectly satisfied with his explanation. We were not aware, at the time we noticed the mistake, that the proprietors of this Journal had written to him on the subject; otherwise we should not have noticed it. More importance has been given to the circumstance than it deserves."

The Editor of the Edinburgh Journal of Medical Science, as will be observed, had promised to make the "*amende honorable*," and accordingly on the 1st of January it appeared under the *appropriate* head of "Practice of Surgery:"—

"The November Number of the London Medical and Physical Journal was announced in the Morning Chronicle of 27th October last by a long advertisement, concluding with a remark, that the two surgical cases referred to Mr. Charles Bell and Mr. John Shaw, in *our* last Number, were cases 'which, in justice, the northern Editor ought to have acknowledged.' We do *acknowledge* (what we suppose the publisher means) that we neglected to mark upon them the words 'Medical and Physical Journal;' and sorry we be that Mr. Souter has taken so small a matter so much to heart, and beg leave very sincerely to assure him, that we will *give him a lift some day or other, by way of compensation, that will make that very heart of his sing for joy within him.* We wrote his 'proprietors' privately to the same effect on the 29th of October last, on receiving rather a bristly epistle from their collective wisdoms. We repeat it, we were as sorry *as if we had trod unintentionally upon their toes.* But, on opening the 'Yellow Book' for the month, *what a yelping, and fuss, and storm in a coffee-pot, presented itself!!!* No less than Plagiarism! a regular hue-and-cry after a 'Literary theft,' as Johnson hath it, 'an adoption of the thoughts and words of another.' Now, we ask, Who is that other? Is it Mr. John Souter, or Dr. Roderick Macleod? Or, are not rather the thoughts and words, simple and all as they are, the thoughts and words of Mr. Charles Bell and Mr. John Shaw, whose names stand attached in our Journal to their respective labours? We would like to hear how the *Editor of the 'Yellow' will get out of this scrape,* in his court of conscience, of having accused us of a crime which it was impossible we could commit."

This paragraph is written in such a temperate and gentleman-like manner,—so entirely devoid of anything bordering upon vulgarity, that we shall not run the risk of diminishing its effect by any comments of our own: we have, however, ventured to put in italics those passages which have most excited our admiration. There is much more in the same style, in which the learned Editor in question has drawn inferences without much regard to the premises. Among other things, he has accused us of "*malicious and designing falsehoods,*" but without stating in what these consist. We prefer an opposite course, stating facts, and leaving others to draw conclusions.

1*st Assertion.*—In the note given above, it is stated that the papers taken from us "are curtailed and reduced," or, as he more politely expresses in his January Number, "licked into shape," for insertion in the Edinburgh Journal of Medical Science.

1*st Fact.*—Mr. Bell's paper is given word for word, without any alteration whatever; in Mr. Shaw's one word is altered (viz. *is* converted into *was*), and the description of a plate of Tiedemann's omitted, because the engraving was not given in the Edinburgh Journal. Every word of Mr. Shaw's is inserted without other change than the solitary one just mentioned.

2*d Assertion.*—"He" (viz. the unfortunate Editor of the Medical and Physical Journal) "had our written apology for the omission in his pocket before the day of publication."

2*d Fact.*—Let the reader cast his eye over the date of our friend's apology, and judge whether we could have been much influenced by the contents of an epistle which, in course of post, could not reach us till two days after our Journal had been in the shops of all the medical booksellers in London. It was indited at No. 62, South Bridge-street, Edinburgh, on the 29th of October, when our November Number was already completed; and that we had not received it was obvious from the notice in our Number for December.

Finding that our papers were so unceremoniously appropriated, we looked more

1

narrowly into the other articles in the *Original* Department of our neighbour, and stated that he borrowed very freely from others as well as from us. To this he indignantly replies, " Without speaking of our present Number, of which we cannot but be vain, our ' Original Department' previously contains exactly seventy-eight papers, of which the account stands thus: Sixty-two original papers, *twelve translations, and four taken from other works.*" Thus, by a quibble as contemptible as that about the word plagiarism in the preceding paragraph, he would have his readers believe that he has altogether taken only four papers from other Journals, and enlisted them in that department of which he is so " vain." But if his translations are not from " other works," whence do they come? The Fourth Number of his Journal alone contains, besides the two he took from us, five other papers taken from " other works," viz. one by Dr. Christison, one by Dr. Gairdner, one by Mr. Hamilton, one by Dr. Bobillier, and one by Professor Dugès; and, not satisfied by swelling this department from these various sources, he has given three reviews under the head of " Original Papers," viz. 1. Various Works on the Arteries; 2. Dr. Elliotson's Paper on the Subcarbonate of Iron; and 3. Dr. Christen's History of Opium. This, we acknowledge, is fully entitled to the appellation of *original,* being quite novel in the history of medical journals; indeed we can only account for this by regarding it as a circumstance, the " control over which was for the time delegated to the printer's foreman." We advise our northern contemporary to let this happen as seldom as possible.

So much for the correspondence between the assertions of this individual and the plain unvarnished facts. It is not our custom to thus dissect the productions of our contemporaries; and if in this instance we have made "the galled jade wince," be it remembered the Editor of the Edinburgh Journal of Medical Science has brought this *exposé* upon himself by the intemperate article alluded to. From the specimens we have given of the manner in which the Edinburgh Journal has thought fit to apologise for an act of piracy too obvious to be denied, we trust our readers will be convinced that if we decline hereafter to answer attacks from the same quarter, it will be from regarding them as unworthy of notice. *Ohe jam satis.*

### METEOROLOGICAL JOURNAL,

*From December 20th, 1826, to January 20th, 1827.*

By Messrs. Harris and Co. Mathematical Instrument Makers, 50, High Holborn.

| December | Moon. | Rain gauge. | Thermom. | | | Barometer. | | De Luc's Hygrom. | | Winds. | | Atmospheric Variations. | | |
|---|---|---|---|---|---|---|---|---|---|---|---|---|---|---|
| | | | 9 A.M. | MAX. | MIN. | 9 A.M. | 10 P.M. | 9 A.M. | 10 P.M. | 9 A.M. | 10 P.M. | 9 *a.m.* | 2 *p.m.* | 10 *p.m.* |
| 20 | | | 42 | 43 | 38 | 29.79 | 29.42 | 93 | 95 | E | SE | Foggy | Cloudy | Rain |
| 21 | | | 39 | 41 | 33 | 29.56 | 30.00 | 91 | 85 | NNW | N | Cloudy | Fine | Fair |
| 22 | ☾ | | 35 | 42 | 42 | 30.14 | 30.11 | 83 | 94 | NNW | WSW | Foggy | Fair | Cloudy |
| 23 | | | 45 | 48 | 45 | 30.12 | 30.16 | 98 | 97 | W | W | — | Cloudy | — |
| 24 | | | 47 | 52 | 45 | 30.17 | 30.19 | 98 | 89 | WNW | NNE | Rain | — | — |
| 25 | | | 46 | 46 | 42 | 30.22 | 30.25 | 96 | 98 | NNE | NE | Cloudy | — | — |
| 26 | | | 43 | 45 | 41 | 30.32 | 30.35 | 96 | 92 | NE | NE | — | — | — |
| 27 | | | 42 | 45 | 34 | 30.41 | 30.43 | 89 | 85 | N NE | NNE | Fair | Fair | Fair |
| 28 | ● | | 35 | 40 | 34 | 30.39 | 30.37 | 85 | 89 | NW | NW | — | — | — |
| 29 | | | 41 | 47 | 44 | 30.28 | 30.17 | 95 | 89 | WNW | WNW | Foggy | — | — |
| 30 | | | 45 | 49 | 42 | 30.10 | 30.11 | 89 | 93 | WNW | WNW | Fair | Fine | Fine |
| 31 | | | 45 | 49 | 45 | 30.09 | 30.01 | 96 | 90 | W | W | — | Fair | Fair |
| Jan. | | | | | | | | | | | | | | |
| 1 | | | 47 | 49 | 39 | 29.82 | 29.43 | 90 | 93 | W | WNW | — | — | Rain |
| 2 | | | 39 | 43 | 25 | 29.42 | 29.48 | 87 | 90 | W | WNW | — | — | Fair |
| 3 | | | 26 | 29 | 18 | 29.41 | 29.40 | 81 | 74 | W | W | — | — | Fine |
| 4 | | | 23 | 30 | 28 | 29.58 | 29.70 | 74 | 90 | W | N | — | Some S. | Some S. |
| 5 | ☽ | | 29 | 30 | 28 | 29.97 | 30.18 | 94 | 87 | N | NNW | — | Fair | Fair |
| 6 | | | 29 | 36 | 37 | 30.16 | 30.08 | 88 | 97 | N | SW | — | Sleet | Sleet |
| 7 | | | 42 | 46 | 42 | 30.00 | 29.96 | 98 | 98 | SW | WSW | Rain | — | — |
| 8 | | | 49 | 50 | 38 | 29.87 | 29.67 | 98 | 98 | WSW | W | Cloudy | Cloudy | Cloudy |
| 9 | | | 46 | 51 | 42 | 29.79 | 29.78 | 86 | 86 | W | WNW | Fair | Fair | Fine |
| 10 | | | 45 | 49 | 34 | 29.70 | 29.34 | 85 | 80 | WSW | W | — | Rain | — |
| 11 | | | 36 | 49 | 34 | 29.23 | 29.23 | 88 | 91 | W | WSW | Rain | — | — |
| 12 | | | 38 | 40 | 31 | 29.37 | 29.83 | 90 | 90 | W | W | Fair | Fair | — |
| 13 | ○ | | 34 | 47 | 46 | 29.74 | 29.66 | 90 | 90 | WSW | SW | — | Rain | Cloudy |
| 14 | | | 51 | 53 | 33 | 29.29 | 29.65 | 84 | 76 | SW | W va. | Cloudy | Fair | Fine |
| 15 | | | 34 | 38 | 34 | 30.04 | 30.16 | 76 | 76 | NWva | W | Fair | — | — |
| 16 | | | 43 | 48 | 36 | 29.92 | 29.90 | 91 | 97 | W | WNW | Cloudy | Rain | Rain |
| 17 | | | 38 | 41 | 35 | 30.13 | 30.15 | 85 | 86 | N | NNW | Fair | Fair | Cloudy |
| 18 | | | 37 | 38 | 32 | 30.15 | 30.16 | 89 | 95 | ENE | NNE | Cloudy | — | — |
| 19 | | | 34 | 38 | 25 | 30.16 | 30.20 | 85 | 96 | ENE | E | — | — | — |

The Rain-gauge having frozen, no account was taken of the quantity of Rain fallen.

